# On the effects of temperature and pH on tropical and temperate holothurians

**DOI:** 10.1093/conphys/coab092

**Published:** 2021-12-15

**Authors:** Enrique González-Durán, Álvaro Hernández-Flores, Maren D Headley, José Duarte Canul

**Affiliations:** 1 Facultad de Ciencias Químico Biológicas, Universidad Autónoma de Campeche, Avenida Ing. Humberto Lanz Cárdenas y Fraccionamiento Ecológico Ambiental Siglo XXIII, Colonia, Ex Hacienda Kalá, C.P. 24085, San Francisco de Campeche, Campeche, Mexico; 2 Universidad Marista de Mérida, Periférico Norte Tablaje Catastral 13941, Carretera Mérida-Progreso, C.P. 97300, Mérida Yucatán, México; 3 Caribbean Regional Fisheries Mechanism Secretariat, Princess Margaret Drive, Belize City, P.O Box 642, Belize

**Keywords:** temperature, sea cucumber, population, physiology, pH, Allee effect

## Abstract

Ocean acidification and increased ocean heat content has direct and indirect effects on marine organisms such as holothurians (sea cucumbers) that are vulnerable to changes in pH and temperature. These environmental factors have the potential to influence organismal performance and fitness at different life stages. Tropical and temperate holothurians are more vulnerable to temperature and pH than those from colder water environments. The high level of environmental variation observed in the oceans could influence organismal responses and even produce a wide spectrum of compensatory physiological mechanisms. It is possible that in these areas, larval survival will decline by up to 50% in response to a reduction of 0.5 pH units. Such reduction in pH may trigger low intrinsic growth rates and affect the sustainability of the resource. Here we describe the individual and combined effects that temperature and pH could produce in these organisms. We also describe how these effects can scale from individuals to the population level by using age-structured spatial models in which depensation can be integrated. The approach shows how physiology can improve the conservation of the resource based on the restriction of growth model parameters and by including a density threshold, below which the fitness of the population, specifically intrinsic growth rate, decreases.

## Introduction

Marine organisms as we know them today are the result of a natural selection process that has existed during different evolutionary periods (Reich, 2010). Unlike the modifications that occurred over thousands of years, the responses observed in the past century and that continue to occur as the temperature increases and the pH of the oceans decreases are taking place in a relatively short period of time (IPCC, 2011; Murray *et al.*, 2013; Widicombe and Spicer, 2008). Thus, there is a need to better understand the physiological adaptations that could allow organisms to adjust to these conditions and how these would, in turn, affect individual survival and the population.

Sea cucumbers are found in virtually all oceans, from the Arctic to the tropics. Purcell *et al.*, (2016) mentioned that there are more than 70 species commercially exploited in the world, and the compilation of field records by Shiell (2004) shows the presence of 28 species, distributed in 8 Fishing areas of the Food and Agriculture Organization (FAO). In the Eastern Center Pacific, 10 commercially important species have been reported (*Astichopus mauritiana*, *Astichopus echinites*, *Holothuria leucospilota*, *Holothuria scabra*, *Holothuria fucogilva*, *Holothuria nobilis*, *Holothuria atra*, *Stichopus hermanni*, *Stichopus chloronotus* and *Thelenota ananas*); in the Eastern Central Atlantic, 4 commercially important species have been documented (*Astichopus multifidus*, *Actinopyga agassizzi*, *Holothuria mexicana* and *Isostochopus badionotus*); in the Central Pacific East, there are records for 2 species (*H. atra* and *Isostichopus fuscus*); and in the Eastern areas, 2 species have also been reported (*H. scabra* and *S. chloronotus*). These species represent examples of sea cucumbers from tropical waters. The temperate water sea cucumbers are located in the Southwest and Northwest Pacific, in fishing areas where the presence of *H. scabra* and *Apostichopus japonicus* have been documented. The cold-water species occur in the Northwestern Atlantic, in areas where the presence of *Chiridota laevis*, *Cucumaria frondosa* and *Psolus fabricii* have been reported (Shiell, 2004). Species of temperate waters such as *H. scabra* and *A. japonicus* are distributed in shallow areas, where temperature and salinity can oscillate rapidly between 20°C and 30°C and fall to 20 USP in rainy season (Hu and Li, 2010). Yang *et al.* (2005) and Yuan *et al.*, (2009) reported having collected juveniles of *A. japonicus* during the winter season at temperatures of 5°C, while An *et al.*, (2009), Dong *et al.*, (2006) and Dong and Dong (2006) reported to have found them at 15°C. Tropical species such as *I. badionotus* and *H. glaberrima* (Pérez-Vega *et al.*, 2013 and Quiñones *et al.*, 2002, respectively) have temperature ranges that fluctuate between 22°C and 24°C. The cold water species (*C. frondosa*), which is located in areas where the fluctuations of temperature are more stable, have been reported at between 3 and 300 m deep (Kale *et al.,* 2013). The wide range of thermal tolerance that some temperate (*A. japonicus* and *H. scabra*) and tropical (*I. badionotus*, *H. mexicana*, *I. fuscus*) sea cucumbers species present, contrasts with the tolerance of cold-water organisms (*C. frondosa*). Without distinction of its distribution, sea cucumbers, especially those of tropical and temperate areas, have adopted strategies that lead to the development of hypometabolism and involuntary non-pathological response (Gullian, 2013; Gullian & Terrats, 2017; Storey, 2015). Hypometabolism occurs by reduction of the organisms’ aerobic scope, which in turn determines physiological responses that generate dormancy and aestivation (Asha & Muthiah, 2005; Morgan, 2008; Yuan *et al.*, 2009; Quiñones *et al.*, 2002). Involuntary non-pathological responses, such as skin ulceration and evisceration, occur as extreme control mechanisms that seek to reset organismal physiological conditions at the expense of anatomical modifications (Garcia-Arraras and Greenberg, 2001; Quiñones *et al.,* 2002; Zamora & Jeffs, 2012).

For the specific case of temperate and tropical sea cucumbers, which is the focus of this work, published studies indicate that these organisms are highly vulnerable to decreases in pH and increases in temperature, especially during their larval stages (Brander, 2010; Morgan, 2008; Yuan *et al.,* 2015). The pH and the water temperature generate diverse biochemical and physiological adaptations in these organisms (Gullian, 2013; Gullian & Terrats, 2017; Wu *et al.,* 2013; Zamora & Jeffs, 2012) that can change the population abundance and density (Brierley & Kingsford, 2009; Doney *et al.*, 2009; Widicombe and Spicer, 2008). Some of these effects are considered to be direct, while others include interactions at the population level (Brierley & Kingsford, 2009; Doney *et al.,* 2009). Reduction of density in these organisms is critical, as they are gonochoric sedentary species. Their reproductive success depends largely on their gregarious behaviour, spawning synchrony and their chemical communication (Fujiwara *et al.*, 2010; Hamel & Mercier, 1996, 1999; Zacarías-Soto *et al.*, 2013). A reduction in density can affect fertilization and thus produce changes in the population intrinsic growth rate (González-Durán *et al.*, 2018; Hutchings, 2014; Kuparinen & Hutchings, 2014). From this perspective, we explore the responses that tropical and temperate sea cucumbers exhibit throughout their life cycle when exposed to variation in water temperature and pH. Our aim is to identify the ways in which changes in pH and temperature influence holothurians at various critical stages and generate hypotheses to explain these observations. In order to do that, we describe the sublethal effects of pH and temperature on holothurian fitness and how this is reflected at the population level. We conclude by suggesting how these threats can be incorporated into population models. The paper ultimately seeks to demonstrate the importance of considering links between environmental conditions and organismal physiology to inform the management of holothurians.

## Responses throughout the life cycle

Temperate and tropical sea cucumbers undergo a series of anatomical modifications that allow them to move from planktonic to benthic lifestyles, to defend themselves against predators and to survive adverse environmental conditions (Hamel & Mercier, 1996). Despite the clear anatomical differences among these stages, the occurrence of evisceration and autotomy in both adults and juveniles, reinforces the hypothesis that the mechanisms involved in temperature perception are similar to those observed in larval stages. In juveniles and adults, the detachment of the sensory fibres of collagen from the body walls (Ferguson, 1982, García-Arrarás and Greenberg, 2001, Wu *et al.,* 2013) suggests that coelomocytes play a greater role in perception. The mechanism is similar in larvae, where all sensitivity depends on coelomocytes (Ferguson, 1982). A plausible mechanism that explains how coelomocytes might respond to temperature could involve the adjustment of their affinity to Ca^2+^, which activates transient receptor proteins, an important kind of membrane protein that constitute a primary mechanism for detecting heat (Crockett and Landroville, 2006; Takahashi *et al.*, 2011). Even when it is very possible that this mechanism could be present throughout the entire life cycle, some differences related to the ontogeny of the stages need to be considered to understand the direct and indirect effects that temperature and pH generates in these organisms.

### Larvae

Some species of sea cucumbers have an indirect development, which means that there are multiple stages of larvae before settlement. In larvae, the effects of temperature are direct and indirect; the former affects the metamorphosis and the development time, while the latter determine the abundance and quality of the food consumed by the organisms (Asha & Muthiah, 2005; Brander, 2010; Yuan *et al.,* 2015). A greater displacement from the optimal value of temperature delays gastrulation, which is the stage in which the digestive tract develops and the feeding activity begins (Smiley, 1986). Greater displacement might limit energy use, resulting in higher physiological costs and slower development, which could increase predatory risk (Dorey *et al.*, 2013). Larvae can delay their development for a few days depending on the availability of food; therefore, food-poor environments can exacerbate their phenotypic plasticity and negatively impact on their survival (Brander, 2010). When analysing the effects of food abundance on auricularia of *Apostichopus mollis*, Morgan (2008) found that food concentration was a major determinant in larvae development. The effect that different feeding rates (6000, 3000, 600 and 300 cells of *Chaetoceros muelleri* ml day^−1^) had on growth was evidenced by smaller larvae in extreme densities and by a better use of food in intermediate levels. These differences could be related to the development of the digestive tract and the size of the mouth, which were longer and bigger, respectively, for larvae that fed in intermediate densities (Morgan, 2008). The viability of the larvae became less evident in organisms that fed 600 and 300 cells ml day^−1^, suggesting that an early application of natural selection filters, in this case represented by food availability, encouraged a better development and magnified the direct environmental effects.

With respect to pH, several studies conducted with larvae indicate that it is a critical factor (Asha & Muthiah, 2005; Hamel & Mercier, 2008; Yuan *et al.,* 2015). An experiment with *Holuthuria spinifera*, which maintained auricularia larvae for 12 days at pH values ranging from 6.5 to 9.0, showed that at pH 7.8 the larvae developed faster, grew better and had better survival rates. Additionally, of the total analysed variables (temperature, salinity and pH), larval development and survival were most sensitive to pH, given the small optimal range (Asha & Muthiah, 2005). The experiments showed that extreme alkaline values (pH 9.0) generated malformation and disintegration of individuals, while the reduction of 0.5 pH units from the optimum (pH 8.0) reduced survival rates by 49%. Recently, Yuan *et al.* (2015) determined the effects that different pH levels (from 7.42 to 8.04) exerted on the post-fertilization success and growth rate of the larval stages of *A. japonicus*. The success of fertilization decreased linearly with the reduction of pH, which according to Stumpp *et al.*, (2011) could be the consequence of the following: (i) high energy cost associated with the regulation of the redox environment and (ii) lack of feeding activity during larval development. In any case, the consequences of pH on the survival of larvae were also related with direct and indirect effects.

### Juvenile and adults

In post-settlement juveniles and adults, pH and temperature most commonly affect growth rates (Dong *et al.,* 2006; Yuan *et al.,* 2009), hypometabolic responses (Storey, 2015; Yuan *et al.,* 2009), aestivation and dormancy (Gullian, 2013; Yang *et al.,* 2006), evisceration (Garcia-Arraras and Greenberg, 2001) and detachment of the body wall (Zamora & Jeffs, 2012). Despite the range of responses, the net effect exerted by the alteration of pH and temperature on the survival of the juveniles is less than those observed in the larvae.

Juvenile sea cucumbers respond to suboptimal temperatures by altering their metabolic activity (Yuan *et al.,* 2009). In *A. japonicus*, temperatures close to the optimum (15.5°C) produced better growth (Dong *et al.,* 2006; Yuan *et al.,* 2009), while higher or lower temperatures decreased food intake, increased metabolism and reduced growth (Zamora & Jeffs, 2012). The main problems associated with suboptimal temperatures are not only tolerance and metabolic energy, but also an increase of diffusion of oxygen and rise of oxygen demands for the maintenance of biological processes (Pörtner & Knust, 2007). Survival at low oxygen levels might be possible through accumulation of high concentrations of CO_2_ in tissues. This could be achieved by compensation from buffers obtained from the surrounding water, food and their spicules (Occhipinti & Boron, 2015; Pörtner *et al.*, 2004). In sea cucumbers a mechanism could decrease the dissociation of Ca^+2^ from metalloproteins and reduce eviscerations, in a similar way for decapod crustaceans (Pörtner *et al.,* 2004). This response constitutes a mechanism that stops unnecessary exposure to adverse circumstances (Quiñones *et al.,* 2002), but it is not the only form in which sea cucumbers defend themselves against the exposure to adverse environmental conditions. In addition to evisceration, sea cucumbers can develop aestivation when exposed to hostile parameters. Yang *et al.* (2006) found aestivation of mature *A. japonicus* at 20°C, while immature organisms aestivated at 25°C. This difference suggests that temperature reduction causes hibernation and that the presence of endogenous reserves, such as that contained in the gonad, satisfies the energy demands to allow the change. The mechanisms that allow restoration after evisceration and aestivation have been investigated by diverse authors. Zhou *et al.* (2014) identified the factors responsible for the growth of the intestine of *Stichopus japonicus* and found the presence of metalloproteins with a maximum activity at pH 5.0 and 50°C. Quiñones *et al.* (2002) studied the interaction of the epithelial and connective tissues of the extracellular matrix during the process of regeneration of the digestive tract and associated the decrease of collagen fibres with the presence of metalloproteins. These studies concluded that the actions of metalloproteins are effective in reducing the formation of collagen in the intestine, which allows the extracellular matrix to re-establish and retain the mesenteries that support the digestive organs again (Quiñones *et al.,* 2002; Zhou *et al.,* 2014). The authors also indicated that the activity of the metalloproteins were related to the concentration of dissolved oxygen (Quiñones *et al.,* 2002; Wu *et al.,* 2013; Zhou *et al.,* 2014), rather than temperature and pH.

To differentiate evisceration from aestivation, we need to consider that the first develops quickly and produces drastic anatomical changes, while the second develops over longer time intervals and does not produce radical anatomical alterations (Du *et al.*, 2013; Gullian, 2013; Yang *et al.,* 2006). To reverse aestivation, it is necessary to carry out a metabolic adjustment that involves antioxidant activity (Gullian, 2013; Ji *et al.*, 2008).

Irrespective of the type of anatomical changes, most of the aforementioned modifications in adults and juveniles are consequence of direct impacts. This, however, does not mean that indirect effects cannot occur. Sun *et al.*, (2013) investigated the seasonal changes of food supply in *A. japonicus*. They found that seasonal fluctuations in environmental conditions modified the feeding behaviour of organisms, presumably affecting their physical conditions.

## Organized structural response

Temperature and pH produce molecular and physiological responses that generate stress and may influence survival. As seen during aestivation, these parameters increase the presence of reactive oxygen species (ROS) and produce an imbalance in the proportion of antioxidants, which causes oxidative stress (Handy *et al.,* 2009; Shao *et al.,* 2015). The ability to adjust the production of antioxidant enzymes in response to acute temperature fluctuations to avoid the increase of ROS occurs in all eukaryotic cells, and the cells of sea cucumbers are not exception (Davidson & Schiestl, 2001). In these organisms the response might be regulated through adjustment of electron transport in mitochondria (Handy *et al.,* 2009), with production of superoxide anions (O_2_^-•^) as minor by-products (Davidson & Schiestl, 2001). As high concentrations of ROS modify protein structure, antioxidant enzyme activity regulates their concentration in mitochondria and cytosol (Candas & Li, 2014). Enzymes such as superoxide (SOD), glutathione peroxidase (GPx), catalases (CAT) and thioredoxins-peroxiredoxin (Trx-Prx) participate in the elimination of O_2_^-^ by converting it into hydrogen peroxide (H_2_O_2_) and water (H_2_O) (Tomanek, 2015; Vives-Bauza *et al.*, 2007). Some experiments performed with adult sea cucumber *A. japonicus*, showed that constant increase of temperature from 16°C to 20°C increased the initial SOD activity of the body wall tissues, from 45.2 to 126.5 and 128.2 U mg^−1^ of protein, respectively, while the increase of exposure time to 25°C for 72 h and 168 h did not produce significant changes in antioxidant activity (Shao *et al.,* 2015).

On the other hand, environmental pH also affects the antioxidant activity of aquatic organisms, especially when this is accompanied by changes in temperature (Matozzo *et al.*, 2013; Wang *et al.,* 2009). When the pH is low, the increase in enzyme production could be a mechanism to cope with oxidative stress and prevent deterioration. For example, in the coelomic fluid of *I. badionotus*, GPx activity increased when the pH changed from 8.0 to 7.7 (Gullian & Terrats, 2017). Although the effects of temperature and pH are important in oxidative stress, few studies have addressed the interaction of these parameters. Matozzo *et al.* (2013) reported that gills and digestive gland of *Chamelea gallina* (clam) and *Mytilus gallopronvincialis* (mussel) at a temperature of 22°C displayed higher activity of SOD and GST in pH 7.7 than pH 8.1. Byrne *et al.* (2013) and Gullian and Terrats (2017) reported the physiological responses of two echinoderms (*Heliocidaris tuberculata* and *Isostichopus badioniotus*, respectively) to the combined effects of temperature and pH. From the limited publications, the evidence shows that at least in the short term, the pH rather than the temperature appears to be more important when antioxidant activity is considered.

As the level of organization increases, the combined effect of temperature and pH causes an increase in energy expenditure, which compromises aerobic metabolism and consequently lead to the development of *peius* thresholds (conditions even worse than those produced only by temperature) (Pörtner & Langenbuch, 2005). The evidence reported by different authors indicates that these thresholds are not always reached, for example in some marine invertebrates the development of hypercapnia stimulates their thermal tolerance (Byrne *et al.,* 2013; Kroeker *et al.,* 2014; Lanning *et al.*, 2010; Madeira *et al.*, 2014). It is possible that the mechanisms involved in increasing resistance to acidic conditions may be related to the activity of the transient receptor channels whose action increases the concentration of cytosolic Ca^2+^ according to the temperature. Thus, if Ca^2+^ increases due to temperature, it could be incorporated to compensate the pH balance, making the organism more resistant to hypercapnia as temperature increases. Of course, this is a hypothesis that needs to be tested.

## Incorporation of molecular and physiological responses to pH and temperature to population dynamics

**Figure 1:**
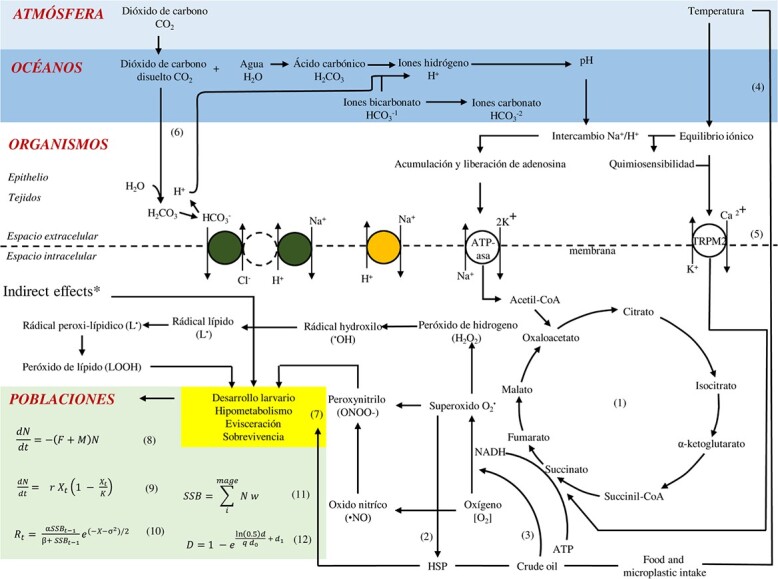
Projection of biochemical pathways and physiological responses into population. Adapted from Seijo *et al.* (1994), Pörtner *et al.* (2004), Pörtner & Langenbuch (2005), Dhaka *et al.* (2006), An and Seijo (2010), Takahashi *et al.* (2011), Kalyanaraman (2013), (Quijano *et al.* (2016) and González-Durán *et al.* (2018). ^*^Although indirect effects constitute an important source of impacts, for the simplicity of the figure they are not considered at this time.

Escalation of molecular and physiological responses that are expected to occur in sea cucumbers as the temperature of the oceans increases and the pH decreases are depicted in Fig. 1. The figure illustrates the production of ROS by mitochondria and the antioxidant response that occurs to reduce the level of reactive species of oxygen and nitrogen (1) (Kalyanaraman, 2013; Quijano *et al.*, 2016). The figure also shows the response of heat shock protein during acute temperature increase (2) (Li C *et al.*, 2021) and the combined effects of co-occurring elevated temperature in the presence of certain contaminants (3) (Li X *et al.*, 2021). As temperature influenced the retention rate of diverse items, such effects are also considered (4) (Iwalaye *et al.*, 2021). The figure shows the influx of calcium ions, which is related to detecting temperatures (5) (Takahashi *et al.,* 2011) and the hypercapnia effects that occur at low levels of pH (6) (Occhipinti & Boron, 2015). Overall, the combined effects modify the physiology and triggers involuntary pathological responses altering the anatomical characteristic of the organisms (Gullian, 2013). Such effects might be lethal in larvae and could impair growth rates in juveniles and adults, compromising reproductive success and reducing recruitment. Sub-lethal effects might include changes in the individual processes such as those described by Pörtner et al. (Pörtner *et al.,* 2004; Pörtner & Langenbuch, 2005), which ultimately affect individual performance (growth, ventilation rate and functional capacity). In the same context, lethal effects could occur when oxidative stress excessively increases hypometabolism, evisceration or detachment of the body wall (7) (García-Arraras and Greenberg, 2001; Gullian & Terrats, 2017; Shao *et al.,* 2015; Zamora & Jeffs, 2012).

Given that some sea cucumber species are commercially exploited, it is necessary to include the impact of fishing mortality (F) in the analysis (An & Seijo, 2010). In this sense, Fig. 1 assumes that the population size depends on the number of individuals that are incorporated into the population in each period as a result of reproduction or, in other words, recruitment to the population (8–9) (An & Seijo, 2010). This biological process can be modelled with a stock-recruitment function (Beverton & Holt, 1957) (10) that includes two parameters and the spawning biomass. Because the model is asymptotic, the maximum number of recruits produced (*a*) is analogous to the population’s carrying capacity. Recruitment to the population depends basically on three factors: the amount of eggs spawned, the fertilization rate and the survival of larval stages (11). Furthermore, since the species have external fertilization, spawners’ density is incorporated as a depensatory function that occurs at low abundance (12) (González-Durán *et al.,* 2018). From this perspective, it is clear that the increase in natural or fishing mortality produces a reduction in abundance, which eventually affects the spawning biomass and reduces the success of recruitment. It is also clear that the effects of temperature and pH are exacerbated when the pressure of the fishing industry is added.

The reduction of density, the main adverse impact that environment produced on sea cucumbers, have been known by several authors as a natural processes of population growth (Hutchings, 2014; Keith & Huchings, 2012; Lierman & Hilborn, 2001). In ecology, such response is known as the Allee effect, which defines a positive relationship between any component of individual fitness and population density (Hutchings, 2014; Stephens *et al.*, 1999). Critical densities below which the collapse of the population is expected have been used to include depensatory mortality in the models of other species (Courchamp *et al.*, 2008; Gascoigne & Lipcius, 2004). Commonly, the incorporation of the Allee effect has been achieved through the addition of exponents in the parameters of the recruitment function (Lierman & Hilborn, 2001); however, the disadvantage is that these exponents do not have a simple biological interpretation (Lierman & Hilborn, 1997). Recently, a model developed for holothurians showed how external factors such as fishing could reduce the spawning stock biomass down to levels, under which reproductive fitness is no longer possible (González-Durán *et al.,* 2018). To include the Allee effect, the authors set the recruitment as a function of density and included an additional term to shift the origin (threshold) to the left; this point represents the moment when all recruitment ceased (12).

Until now, the inclusion of adverse environmental effects has been done considering probable scenarios of change using decision theory and adding stochasticity based on expected environmental trends. Seijo *et al.*, (2016) determined the bioeconomic effect of ocean acidification in the fisheries of calcified species. These authors developed an age-structured dynamic model linked to decision tables with alternative decision criteria in the absence of probability of occurrence of three alternative pH states of nature reported by as climate change scenarios by the Intergovernmental Panel on Climate Change (IPCC). The authors built dynamic functions for natural mortality M (an increasing non-linear function of pH) and K of Von Bertalanffy growth equation (a decreasing non-linear function of pH), dependent on the possible dynamic trajectories of pH reported by IPCC scenarios. Parameter values for the above-mentioned functions used experimental results reported in the literature. Another analysis that has been used to include the effects of environmental variability on fisheries is the sensitivity analysis of reference points. Defeo and Seijo (1999) produced variation of the parameters using the bootstrap technique and subsequently carried out risk analysis according to the maxi-min, mini-max and maxi-max criteria. On the other hand, Hare *et al.*, (2010) developed a coupled climate population model for *Micropongonias undulatus* based on the hypothesis that the recruitment of juveniles was affected by temperature. The authors considered the decadal variation of temperature to simulate the behaviour of 100 hypothetical populations, introducing stochasticity in the M and F parameters, as well as variability in recruitment. Punt *et al.*, (2015) considered different levels of tolerance to ocean acidification and developed pre- and post-recruitment models for *Chionoecetes bairdi*. These authors used a stage-structured population model to forecast the change over time in recruitment to the first size class in the post-recruitment model; in this model, recruited male were modelled and their biomass was used as a proxy for fertilized egg production. The approach was similar to the vector tracking projections carried out by Seijo *et al.* (2016). Co *et al.* (2015) linked three models (biogeochemical, biological and socioeconomic) into an integrated assessment model that simulate oceanographic and population dynamics and the socioeconomic relationship for the fishery of *Placopecten megallanicus*. Several studies conclude that ocean acidification will have negative consequences on exploited wild populations.

The studies summarized above reveal how the environment could be considered when estimating population growth responses. Doing so has the potential to illustrate the importance of physiological tools and modelling for exploring different environmental scenarios. From this perspective, a complete understanding of the life cycle of the species including its critical stages, as well as the recognition of the demographic patterns that define its distribution and fitness and the identification of appropriate procedures to include responses at the population level, are fundamental aspects to enhance the conservation of the resource.

## Conclusions

Given the importance of pH and temperature for the survival of many marine populations, it is extremely important to understand the mechanisms that allow them to cope with changes in these parameters and develop logical procedures to expand these responses. In temperate and tropical sea cucumbers, the direct and indirect effects of high variation of pH and temperature result in sublethal and lethal responses, depending on the stage of their life cycle. The critical stage of holothurians to the adverse effects of temperature and pH appears to be the recruitment phase. In larvae, a pH reduction of 0.5 units can lead to elevated mortality with the potential to impact population growth. On the other hand, juveniles and adults display a wider spectrum of responses in some cases associated with anatomical modifications. Thus, the mechanisms that define holothurian responses to changes in pH and temperature occur at different biological levels of organization, from the molecular to the entire organism. In this way, the responses can be incorporated at the population level through the parameters of the population model equations and create realistic scenarios associated with different climate change projections.
